# The R-Operon: A Model of Repetitive DNA-Organized Transcriptional Compartmentation of Eukaryotic Chromosomes for Coordinated Gene Expression

**DOI:** 10.3390/genes7040016

**Published:** 2016-04-22

**Authors:** Shao-Jun Tang

**Affiliations:** Department of Neuroscience and Cell Biology, University of Texas Medical Branch, Galveston, TX 77555, USA; shtang@utmb.edu; Tel.: +1-409-772-1190

**Keywords:** chromosome, chromatin, transposon, repetitive DNA, DNA repeat, operon, transcription, gene expression, transcription factory

## Abstract

In eukaryotic genomes, it is essential to coordinate the activity of genes that function together to fulfill the same biological processes. Genomic organization likely plays a key role in coordinating transcription of different genes. However, little is known about how co-regulated genes are organized in the cell nucleus and how the chromosomal organization facilitates the co-regulation of different genes. I propose that eukaryotic genomes are organized into repeat assembly (RA)-based structural domains (“R-operons”) in the nuclear space. R-operons result from the interaction of homologous DNA repeats. In an R-operon, genes in different loci of the linear genome are brought into spatial vicinity and co-regulated by the same pool of transcription factors. This type of large-scale chromosomal organization may provide a mechanism for functional compartmentation of chromosomes to facilitate the transcriptional coordination of gene expression.

## 1. Introduction

It is clear now that biological functions in the cell are controlled by multiple functionally related proteins. Initiating biological functions requires the co-expression of their correspondent genes. Thus, achieving the coordinated expression of functionally related genes is a fundamental biological process. In prokaryotic cells and viruses, the co-regulated expression of functionally related genes is often achieved in operons, in which genes are organized in the same transcriptional unit (operon), and the expression of these genes is controlled by a shared promoter (operator) [1]. Although this format of co-regulation is maintained in a small number of eukaryotic organisms (e.g., *Caenorhabditis elegans*), operon-like transcriptional units are not found in most eukaryotic organisms. Instead, co-regulated genes in eukaryotic genomes are often dispersed in different loci, and their co-regulation is predominantly achieved by employing the same (or similar) *cis*-regulatory elements (e.g., enhancers) that respond to the same trans-acting transcription factors.

More recent exciting findings reveal that transcription in the eukaryotic nucleus is compartmented into specific domains called transcription factories (TF) [2] in which genes undergoing transcription may share the same pool of transcriptional machinery. Because of the large number of genes expressed in any specific type of cell in higher organisms, each TF must host the transcription of multiple genes. A major mystery of which, to date, we know little is what causes gene compartmentation in TFs. One simple assumption is that the formation of TFs is a direct consequence of co-transcription. Under this scenario, the enhancer-promoter interactions mediated by trans-acting transcription factors may play important roles in TF formation. Consistent with this idea, specific DNA-binding transcription factors such as CTCF have been suggested to be critical for TF formation [3].

Here, I would like to consider the intrinsic genomic organizations that may contribute to TF formation and transcription co-regulation. My basic assumption is that, given the importance of gene co-regulation and TF formation, the organization of chromosomes is likely evolved in such a way as to facilitate these processes. However, little is known about this aspect of functional genomic organization. I put forward here a potential chromosome organization mechanism that may critically contribute to TF formation and gene co-regulation in the nucleus of higher organisms.

The core idea of the hypothesis is that DNA repeat-directed compartmentation of chromosomes generates a structural platform that facilitates TF formation and gene co-regulation. I have recently proposed a repetitive DNA-based principle of chromosome organization, named the CORE (chromosome organization by repetitive elements) theory [4] and postulated the structural features of repeat-formed skeletons of mitotic and interphase chromosomes [5,6]. The chromosome CORE theory states that in the nucleus DNA repeats tend to associate with their homologous sequences by repeat pairing (RP) to form repeat assemblies (RAs) and that RP provides a driving force to fold or crosslink chromatins in a site-specific manner [4]. As such, DNA repeats function as cis organizer modules for chromatin organization in chromosomes. This organization may have important functional consequences because it provides a structural mechanism to compartment genes at different genomic loci into the same nuclear domain. From this perspective, I want to examine the implication of this postulation in gene co-regulation.

## 2. A Structural Model of Repeat-Coordinated Gene Co-Regulation: The R-Operon

How can transposon-like dispersed repetitive DNA coordinate the expression of a set of genes? One emerging model is that the genes are controlled by *cis* elements, including promoters and enhancers, in the transposon [7,8,9]. In this manner, the transcription of these genes is coordinated by the same (or similar) cis elements. Conceptually, this model is the same as the traditional idea that co-regulated genes are controlled by the same (similar) *cis*-elements and is not the focus of this discussion. Here, I want to consider a novel conceptual framework that emphasizes the role of DNA repeats in gene co-regulation from the perspective of chromosomal organization. This organization-based viewpoint may provide a new mechanism by which DNA repeats co-regulate genes, in addition to the promoter-based model mentioned above.

**Figure 1:**
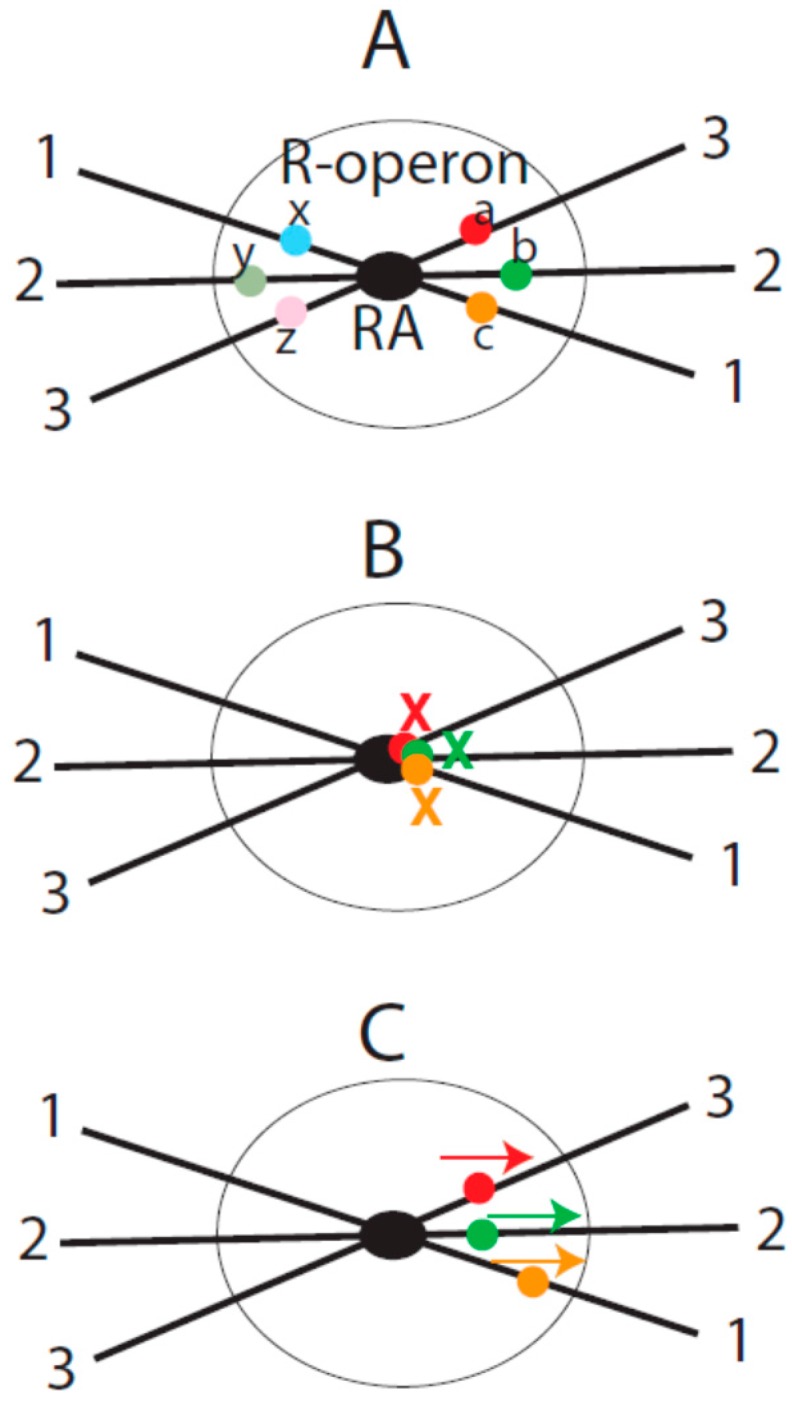
Coordination of gene expression by the R-operon. (**A**) Conceptual illustration of R-operon. Shown is an R-operon consisting of a repeat assembly (RA) from three genomic loci (indicated by 1, 2 and 3) and the associated genes (colored dots) proximal to the RA (black dots); (**B**) Co-repression in the R-operon. When genes are closely associated with the RA, their transcriptional activity can be suppressed (see text); (**C**) Co-activation in the R-operon. Genes proximal to the RA may be co-activated by trans-activation or chromatin contacts facilitated by the RA (see text).

The central idea of the current model is that RAs organize their associated genes into transcriptional units or domains in the nucleus. I consider such structural and functional units to be a new genetic entity that is between the levels of genes and genomes and name this entity repeat-organized operon (R-operon). An R-operon contains an RA and RA-associated genes (including related *cis*-elements) (Figure 1A). Genes in the same R-operon may be co-transcribed by the same pool of transcription factors in this domain. It is probable that an R-operon constitutes a TF or a domain of a TF. In the proposed mesh structure of interphase chromosomes [6], R-operons would correspond to the chromosome nodes and their associated genes in the mesh.

Unlike the classical operons in prokaryotic organisms, the novel feature of the R-operon model is that genes in an R-operon often, if not always, are not at the same genomic locus. Instead, they are organized around RAs in the 3D nuclear space. In addition, an R-operon is a transient chromatin organization. Its assembly and disassembly is controlled by dynamic RPs, which are modulated by the physiological state of a cell [4].

The idea of RPs and RAs appears to be consistent with a large amount of empirical data that I have discussed before [4,5,6], as well as findings that have emerged more recently [10,11,12]. The RA formation-caused clustering of dispersed repeats would undoubtedly bring the genes that are close to individual repeats at different genomic loci into the same spatial domain in the nucleus. Therefore, it is self-evident that the formation of RAs of dispersed repeats will cause structural compartmentation of genes in the nuclear space. I envision that such structural compartmentation may facilitate co-regulation of the involved genes.

Because different RPs likely have different stabilities in a given cell state [4], not all DNA repeats will be assembled into RAs. Thus, I imagine that a specific cell state corresponds with a specific set of R-operons. In other words, different cell types of the same organism are biologically specified by different sets of R-operons that are organized by different RAs. As such, an R-operon is an organizational and functional compartment of the genome. Because of the dynamic nature of RPs, most, if not all, R-operons should be considered as transient structures, although different R-operons may have different lifespans. The assembling and disassembling of RAs may provide a driving force for chromatin movement and gene repositioning in the nuclear space. I conceive that the formation of R-operons probably provides an organizational mechanism for coordinating the expression of a large number of functionally related genes that are active (or inactive) in specific tissues or cell types. For example, in one type of human cells, a set of R-operons is in place, which organizes the expression of genes of this cell type, while in another cell type, a different set of R-operons is formed to organize the expression of genes required by the cell type. There may be overlap between R-operons of different cell types. It is common for a given gene to function in distinct cell types of higher organisms. The structural plasticity of an R-operon driven by RPs provides a plausible mechanism for a gene to be co-regulated with different groups of genes in different R-operons so that it can fulfill its biological roles in different types of cells. In this way, the functional compartmentation of a genome can be very plastic, which may have evolved for the formation of numerous cell types in higher organisms. How the RA-organized gene compartmentation is dynamically regulated in a cell is unclear at this stage and is an important problem for future investigation.

The R-operon model predicts that some temporally and/or spatially co-regulated genes may be tagged by related dispersed repeats and that genes tagged by related dispersed repeats will have a higher probability of sharing similar expression profiles. Indeed, analysis of gene expression in mouse oocytes and preimplantation embryos indicated that different transposable elements are associated with synchronous expression of different sets of genes at specific developmental stages [13]. For example, mouse transcript retrotransposon is predominantly associated with genes expressed in oocytes, while MuERV-L retrotransposon is mainly with genes expressed in two-cell stage embryos. In addition, cell type- and tissue-specific transcripts containing LINE L1 repeats have been reported [14]. Recent studies by Huda *et al.* appear to suggest a role of repeated elements upstream of promoters in coordinating the expression of human genes [15]. The model also predicts that genes that are tagged by members of the same repeat family have a higher probability of co-localizing in the nuclear space and sharing similar expression profiles. Conclusive confirmation of such predictions may be obtained from systematic association analysis, which unfortunately is difficult at present due to the lack of required genome-wide data. Nonetheless, currently available technologies such as fluorescent *in situ* hybridization (FISH) and chromosome conformation capture (3C) or circular chromosome conformation capture (4C) could be useful to test these specific predictions. Although this model should be considered unproved yet, I hope that the specific testable predictions will stimulate investigation. Although the current model emphasizes the organizational role of repeats in formation of the R-operon, it does not preclude the possibility that some of the repeats in the R-operon also contain cis elements for transcriptional regulation. In fact, the two mechanisms likely contribute to coordinating the expression of repeat-associated genes. In addition, the transcription of the repeats may impact on the associated genes. For example, it is well established that transposons can generate small RNAs to influence the expression of other genes [16].

## 3. Potential Mechanisms of R-Operon-Based Co-Regulation

In the following sections, I provide a few more illustrations to show how RAs may impact the expression of their associated genes in R-operons. I would like to point out that these postulated scenarios are not meant to exhaust all possible mechanisms that could be employed by repetitive DNA to regulate gene expression. Instead, only potential mechanisms from the perspective of the impact of RA-mediated chromatin organization are considered. In particular, we consider how RA formation may generate a coordinated effect on the transcriptional activity of the associated genes (Figure 1).

As an example, assume that there are three homologous repeats that reside in three different loci (labeled as 1, 2 and 3 in Figure 1) that are far apart from one another in the linear genome. Let us assume that there are two genes (a, b, c, x, y and z in Figure 1A) proximal to each repeat. For simplicity of description, let us consider that there is one gene on each side of the repeat. When the three repeats are assembled into an RA by RP, the genes are brought into proximity in the nuclear space. Now, let us consider the possible scenarios of the transcription co-regulation among these genes.

(1) RA-mediated co-suppression. When the genes are close to the RA, the functions of their promoters and other cis-elements can be masked by the associated RA. This masking effect can result from the physical barrier of the RA or from epigenetic modifications induced by the RA. The consequence of such a masking effect is co-repression of genes (Figure 1B). When the RA is disassembled due to the physiological changes of the cell (e.g., stimulated by external signals), the masking effect of the RA on the genes would disappear. In this situation, the transcriptional repression of the genes would be removed.

Alu repeat-associated co-regulation of miRNA genes is probably consistent with such a postulated scenario. Alu repeats are dispersed in the human genome. Alu-harboring chromatins are poorly accessible to restriction enzymes in HeLa cells [17,18]. Alu transcription is also suppressed in these cells. Stresses (e.g., heat shock and adenovirus infection) induce the accessibility to Alu chromatins in HeLa and 293 cells, which promotes Alu transcription [17,18]. These observations are consistent with the idea of the formation of compact Alu RAs [4]. The RA formation is likely regulated by epigenetic modifications of the repeat [19]. Recent studies provide interesting insights into the impact of Alu repeats on the co-regulated expression of microRNA (miRNA) genes. On human chromosome 19, there is a cluster of miRNA genes (*miR-512-5P*). These genes are interspersed among Alu repeats that function as the promoters of the miRNA genes [20]. DNA demethylation or a histone deacetylase (HDAC) inhibitor, which may promote the disruption of RAs [4], dramatically up-regulated miRNA transcription [21]. Although it remains to be demonstrated, the epigenetically regulated miRNA transcription may be interpreted by the following simple mechanism. Under resting conditions, Alu repeats nearby the microRNA genes are assembled into compact RA, and epigenetic modifications (e.g., DNA methylation) facilitate RA formation or stabilize RAs. In this structure, Alu promoters are masked in RAs, and the transcription of miRNA genes is suppressed. In contrast, DNA demethylation and HDAC inhibition promote Alu RA disruption. Consequently, the Alu promoters are de-masked, and the transcription of the miRNA genes is activated.

(2) RA-facilitated trans-regulation. In another possible scenario, a set of genes could be brought into the same chromosomal domain by the formation of the RA but without a masking effect from the latter because of the relatively distant positions from the RA. Because of their vicinity to each other, the transcriptional activity of one gene may affect the others. For example, the activation of one gene may have a trans-activation effect on the nearby genes around the RA, even though the latter genes may not have the same cis-elements. The trans-effect is dependent on the vicinity of these genes in the nuclear space and is probably caused by sharing the pool of transcriptional factors.

The key to this mode of co-regulation is that the *cis*-element of one gene modulates the transcription of another gene (or genes) on the paired chromatin in *trans*. The genetic phenomenon of the trans-sensing effects (or so called transvection) illustrates this possibility better. Transvection is manifested by the activation (or suppression) of genes on homologous chromosomes by regulatory elements in trans. This genetic phenomenon is well documented in various organisms [22], including plants [23] and *Drosophila* [24]. For example, Mellert and Truman reported that in transgenic flies when two transgenes (LexA or GAL4) with their own regulatory elements are inserted at the same integration site and paired, the enhancer of one transgene can drive or repress expression of the other paired transgene [24]. Because DNA repeats can undergo somatic pairing in the cell [4], I envision that trans-regulation similar to transvection provides a mechanism of DNA repeat-directed coordination of expression of multiple genes in R-operons (Figure 1C).

Can DNA repeats indeed modulate gene transcription in *trans*? Work on the *Drosophila* gypsy repeats provides direct evidence for this possibility. The *Drosophila* gypsy repeats are retrotransposons. Because of their ability to block enhancer-promoter interactions when positioned between them, gypsy repeats are considered to have an insulator activity [25]. The insulator in gypsy consists of a cluster of 12 binding sites for the Su (hw) zinc-finger protein. It has been demonstrated that Su(Hw)-mediated intrachromosomal pairing of gypsy in cis can neutralize its insulator activity [26,27]. Interestingly, pairing between gypsy insulators facilitates the enhancer action in *trans* [28]. The presence of the gypsy insulators in homologous chromosomes, even at a distance of 9 kb downstream from the promoter, markedly improves the *trans* activation of gene *yellow*. Furthermore, gypsy stabilizes *trans* activation between distantly located enhancers and a promoter. These findings clearly indicate that gypsy pairing is involved in communication between loci in *trans*.

(3) RA-facilitated chromatin contact. If there is another set of genes that do not share cis-elements, then their transcription may not be directly coordinated by the interaction of their cis-elements. However, the formation of the RA does bring these genes into the same spatial domain in the nuclear space. This may facilitate chromatin contacts during their transcriptional activation (Figure 1C). The chromatin contact in return may promote the co-regulation of transcription among these genes, as suggested recently [29].

The above postulations only describe some simple scenarios of RA effects on the associated genes. The actual situation may be more complex if genes use multiple *cis*-elements to control their transcription. Nonetheless, the essential point that I wish to make from these illustrations is that the formation (and disruption) of RAs can provide a structural (organizational) means to coordinate the expression of multiple genes, which is critical for the expression of biological functions (e.g., during developmental processes).

## 4. Implications

### 4.1. A novel mechanism for gene co-regulation

Coordination of the activity of functionally related genes is critical for all organisms. Previous considerations on the potential mechanisms underlying the coordination have mainly focused on the role of specific transcription factors that act on the enhancers that are shared by the coordinated genes. According to this traditional view, when a specific transcription factor is expressed, it co-activates (or co-suppresses) the genes that have the binding site of this transcription factor in their enhancers. Here, I propose a novel model for the coordination of the transcription of different genes, from the perspective of DNA repeat-mediated spatial organization of genes in chromosomes in the nuclear space. I consider this spatial organization of genes as a way for functional compartmentation of chromosomes. Under this framework, one can envision that a chromosome contains many functional domains that are organized by RAs (*i.e.*, R-operons). In individual domains, there is an RA and genes around it. These genes are expected to show a degree of coordinated transcriptional activity and may constitute a part of a transcription factory. These RA-organized transcription domains are dynamic. At any given time, some are disappearing due to RA dissociation, while new ones are forming because of the assembling of other RAs. In this way, transient coordination of specific combinations of genes can occur to fulfill specific biological requirements.

### 4.2. A novel mechanism for cell-type specific expression of different target genes of the same transcription factor

One specific transcription factor (e.g., NF-κB and CREB) often has many target genes in the genome. It is well documented that different target genes can be specifically expressed in different cell types. This phenomenon is hard to explain by simply applying the classical idea of transcription factor/cis-element interaction, and requires additional mechanisms in play. Without excluding other possibilities (e.g., different epigenetic modifications of the target genes), I envision that the R-operon model provides an organizational mechanism for such differential regulation to occur. In other words, different target genes of the same transcription factor may be tagged with different repeats that form different R-operons in different cell types, and thus are differentially expressed.

### 4.3. A novel mechanism for functional collaboration of genes

A gene is often expressed in different cell types to execute distinct biological functions. The biological functions of a gene in different cell contexts are determined by its association of expression with a specific set of genes that genetically define the characteristics of the cell. What are the mechanisms by which the co-expression of a set of functionally related genes is determined? Multiple mechanisms are likely involved to control the functional collaboration of genes, because one gene can be within different functional groups in different cell types. As mentioned above, the best-characterized mechanism is the sharing of similar cis-elements for transcription so that the genes can be co-regulated by the same transcription factors. I propose here that the repeat-mediated compartmentation of genes in the nuclear space provides an organizational mechanism for the co-expression of genes. Because of the trans-action of cis-elements described above, this mechanism does not require the sharing of similar cis-elements of the co-expression genes. Since a DNA repeat (e.g., transposon) has the potential to interact with many other homologous repeats, this mechanism generates enormous plasticity of gene collaboration. Under this organizational perspective, a gene can be grouped into different functional sets of genes. In contrast, the cis-element-mediated mechanism does not create such a potential for plasticity. Since RP dynamics are likely modulated by cellular physiology (e.g., ion concentration and the expression of the proteins that regulate RP), RP-coordinated gene co-expression provides a cell context-relevant mechanism for functional collaboration of genes.

## 5. Conclusions

Emerging evidence suggests an important role of repetitive DNA sequences, especially transposons, in regulation of gene transcription. Previous work emphasizes the role of transposons as cis-regulatory elements such as promoters. Here, I propose that repeat paring (RP)-based chromatin interactions provide a mechanism of structural compartmentation that generate functional domains (R-operons) in the chromosomes. In these functional domains, genes that are distant in the linear genome are brought into spatial proximity in the nuclear space and thus can be co-regulated. According to this model, the assembling and disassembling of repeats (*i.e.*, the formation and disruption of RP, respectively) may cause coordinated effects (either co-activation or co-repression) on the transcription of genes proximal to the participating repeats. I propose that repeat assemblies (RAs) provide a structural platform for gene co-regulation and that the regulation of the dynamic formation or disruption of RAs provides a mechanism to coordinate the activity of genes in response to external stimuli or changes in cell states.
